# S100A8/A9 high-expression macrophages mediate renal tubular epithelial cell damage in acute kidney injury following acute type A aortic dissection surgery

**DOI:** 10.3389/fmolb.2025.1530741

**Published:** 2025-04-09

**Authors:** Xiujuan Cai, Xin Li, Jian Shi, Lu Tang, Jie Yang, Ronghuang Yu, Zhigang Wang, Dongjin Wang

**Affiliations:** ^1^ Department of Cardiac Surgery, Nanjing Drum Tower Hospital Clinical College of Nanjing University of Chinese Medicine, Nanjing, China; ^2^ Department of Cardiac Surgery, Nanjing Drum Tower Hospital, Chinese Academy of Medical Sciences & Peking Union Medical College, Nanjing, China; ^3^ Department of Cardiac Surgery, Nanjing Drum Tower Hospital, Affiliated Hospital of Medical School, Nanjing University, Nanjing, China

**Keywords:** S100A8/A9, aortic dissection, acute kidney injury, macrophage, TNF pathway

## Abstract

**Background:**

Acute kidney injury (AKI) is a major complication after acute type A aortic dissection (ATAAD), with an incidence rate of 20–66.7%. Many patients with AKI after ATAAD surgery show no clear signs of ischemia-reperfusion injury. In our previous study, S100A8 and S100A9 were identified as predictive biomarkers of AKI after ATAAD surgery. These proteins are primarily expressed in neutrophils and macrophages, where they contribute to cell damage and immune cell activation. However, the roles of S100A8/A9 in ATAAD-associated AKI remain unclear.

**Methods:**

In this study, transcriptomics sequence was applied to identify differentially expressed genes in renal tubular epithelial cells (TCMK-1), stimulated by culture supernatant of S100A8/A9 overexpressed and downregulated RAW264.7 cells. Single-cell sequencing data were used to identify cell clusters with high S100A8/A9 expression. Cross-analysis between RNA sequencing datasets was used to investigate common pathways enrichment in both *in vitro* and *in vivo* models. Molecular biology experiments were used to explore the downstream signaling pathways of S100A8/S100A9.

**Results:**

We found that S100A8/S100A9 expression levels were increased and co-localized primarily with macrophages in the kidneys of AKI mice. Marker genes of M1-type macrophages, like Nos2 and Il1b, were upregulated in S100A8/A9 overexpressed M1-type macrophages, while the opposite was observed in the downregulated group. In transcription sequencing results, TCMK-1 cells stimulated by the supernatant from S100A8/A9 overexpressed and downregulated RAW264.7 cells can activate the TNF and PPAR pathway respectively. Cross-analysis revealed that the TNF signaling, IL-17 signaling, and other inflammatory pathways were enriched in both S100A8/A9-related renal tubular epithelial cell impairment and other AKI sequencing datasets. Finally, recombinant protein S100A8/A9 activated the TNF signaling pathway in renal tubular epithelial cells.

**Conclusion:**

These findings suggested that S100A8/A9 were promising predictive biomarkers for AKI after surgery for ATAAD. S100A8/A9 were upregulated and primarily localized in renal macrophages, where they promoted the transformation of macrophages into the M1 phenotype. S100A8/A9 overexpressed macrophages activated the TNF signaling pathway through secretion and direct interaction with renal tubular epithelial cells, highlighting the critical role of TNF signaling in AKI after ATAAD surgery.

## 1 Introduction

Acute type A aortic dissection (ATAAD) is an emergency cardiovascular disease characterized by intimal rupture followed by a tear in the aortic media, involving the ascending aorta (Mussa et al., 2016; Evangelista et al., 2018). Acute kidney injury (AKI), marked by a sudden deterioration in renal function, is a common surgical complication following ATAAD surgery (Roh et al., 2012). ATAAD-associated acute kidney injury (ASA-AKI) has a high incidence of 20%–66.7% (Li et al., 2020; Helgason et al., 2021). ASA-AKI patients have higher inhospital mortality, lower long-term survival, an increased probability of other concomitant postsurgical complications, and a heightened risk of progression to chronic kidney disease (Sasabuchi et al., 2016; Arnaoutakis et al., 2023; Kellum et al., 2021). The diagnostic criteria for ASA-AKI, often based on the 2012 Kidney Disease: Improving Global Outcomes (KDIGO) guidelines, are as follows: i) serum creatinine (sCr) level ≥26.5 μmol/L or more than 1.5 times the basal value within 48 h, which clearly or presumably occurs within 7 days of ATAAD operations, and ii) urinary output <0.5 mL/(kgh) for more than 6 h (Author Anonymous, 2012). Clinicians need to recognize and correct risk factors in advance that may affect renal perfusion and cause prerenal azotemia in postoperative ATAAD patients. When ASA-AKI is diagnosed, improving renal perfusion, avoiding nephrotoxic drugs, and using continuous renal replacement therapy for severe cases are employed to alleviate kidney damage. Nevertheless, no specific treatment for ASA-AKI exists yet (Stanski et al., 2023).

The etiology of ASA-AKI remains unclear, but it was strongly associated with risk factors such as poor perfusion due to ATAAD formation, high complexity of aorta repair and replacement surgery, duration of cardiopulmonary bypass, and the use of contrast agents and nephrotoxic drugs (Wang et al., 2022). Although ischemia-reperfusion (I/R) injury is a risk factor for ASA-AKI, our team has observed that a subset of patients who lack the evidence of I/R injury can develop ASA-AKI, and the underlying mechanism for these cases remains unclear (Wang et al., 2020). We further revealed that S100A8 and S100A9 (S100A8/A9) could serve as predictive biomarkers for ASA-AKI, being significantly upregulated in plasma of ASA-AKI patients at 0 h after surgery by proteomic testing (Wang et al., 2023). However, the mechanisms by which S100A8/A9 contribute to ASA-AKI remain undefined.

S100A8 and S100A9 are members of the calreticulin family and ligands for Toll-like receptor 4. These proteins often act as heterodimers involved in inflammatory responses and immunomodulation, predominantly expressed in myeloid cells such as neutrophils and macrophages (Chen et al., 2023). In several inflammation-related diseases, such as myocardial infarction, acute lung injury, acute kidney injury, and cancer, S100A8/A9 were reported to be valuable biomarkers (Wang et al., 2023; Li et al., 2019; Gong et al., 2024; Chen et al., 2024). In single-cell sequencing of AKI mice, Yao et al. (2022) found that S100A9 was predominantly expressed in macrophages, which were detected in kidney sections early at 2 h post-AKI. These macrophages also highly expressed inflammation-related genes such as *Il1b, Tnf, Tnfaip3, Il1r2, Il1rn, Cxcl2, Cxcl3, Ccrl2, Mmp8*, and *Mmp9* (Yao et al., 2022). However, previous studies have reported that neutrophils were the primary source of S100A8/A9 in myocardial infarction and that these proteins affected mitochondrial complex I function, inducing cardiomyocyte apoptosis (Li et al., 2019). In addition, Huang et al. showed that S100A8/A9 were located in Ly6G-positive neutrophils in AKI mice and that the expression of CXCR2 and S100A8/A9 was strongly correlated in the transcriptome of immune cells in AKI (Huang et al., 2023). Excessive immune cell infiltration and activation induce sustained inflammatory responses, primarily affecting renal tubular epithelial cells, which play a pivotal role in inflammation and repair during AKI (Rudman-Melnick et al., 2020). In this study, we further investigated the influence of S100A8/A9 on macrophages and renal tubular epithelial cells in ASA-AKI, which provides potential opportunities for clinical treatment.

## 2 Method and material

### 2.1 Ethical declaration

The animal research project was approved by the Institutional Review Board (Ethics Committee) of the Ministry of Health of the People’s Republic of China and the Animal Protection and Utilization Committee of Drum Tower Hospital affiliated with Nanjing University (Approval number: 2024AE01015).

All experiments were conducted in accordance with relevant guidelines and regulations. The study involving live animals adhered to the ARRIVE guidelines (PLoS Bio 8 (6), e1000412, 2010). Anesthesia and euthanasia procedures for the animals met the requirements outlined in the American Veterinary Medical Association (AVMA) Guidelines for Animal Euthanasia (2020).

### 2.2 Animal experiment

Male C57BL/6 mice (8 weeks old; 23–25 g) used in this study were purchased from GemPharmatech Co., Ltd with a license for the use of laboratory animals [SYXK (SU) 2018-0027] and a permit for the production of laboratory animals [SCXK (SU) 2018-0008]. The mice were housed in a standard environment free from specific pathogenic bacteria, with temperature controlled at 23°C–26°C, humidity maintained at 50%–60%, and a 12-hour circadian rhythm. Adequate feed and water were provided.

The mouse AKI model was established as described by Wei and Dong (2012). All mice were randomly assigned to the AKI and control (Ctrl) groups. Mice in the AKI group were anesthetized by intraperitoneal injection of 1% sodium pentobarbital (50 mg/kg) and then fixed on a thermostatic table. The skin on the back was shaved and disinfected. The skin incisions were made near the right and left subcostal margins of the back. The bilateral kidneys and renal pedicles were bluntly isolated. The bilateral renal pedicles were clamped shut for 30 min using miniature arterial clips, causing the kidneys to gradually darken. After the clips were removed, the color of the kidneys gradually returned. The dorsal surgical incisions were closed layer by layer. A similar surgical procedure was performed in the Ctrl group, but the renal pedicles were not clamped after exposure. The mice were kept warm on a heating pad until they awoke after surgery. At 24, 48, and 72 h after surgery, the mice were euthanized by intraperitoneal injection of 1% sodium pentobarbital (200 mg/kg). The kidneys of all groups were obtained and stored in a refrigerator at −80°C or fixed in 4% paraformaldehyde for at least 48 h.

### 2.3 Cell culture

RAW264.7 (CL-0190, Procell), TCMK-1 (SNL-602, Sunncell), and HEK293T (CL-0005, Procell) were cultured in DMEM (11995065, Gibco) containing 10% fetal bovine serum (10270106, Gibco) and 1% penicillin–streptomycin (450-201-EL, Wisent). Primary mouse renal tubular epithelial cells (mRTECs) (PRI-MOU-00051, ZQXZBIO) were cultured in DMEM/F-12 medium (11320033, Gibco) containing 10% fetal bovine serum and 1% penicillin–streptomycin. All cells were maintained in a constant-temperature incubator at 37°C with 5% CO_2_ and 95% air. When the cells grew to enough density, they were passaged using trypsin-EDTA resolution (25200072, Gibco).

Mouse primary bone marrow-derived macrophages (BMDM) were extracted from the leg bone marrow of 8- to 10-week-old male mice and cultured in RPMI-1640 medium (61870127, Gibco) containing M-CSF (50 ng/mL, SinoBiological), 10% fetal bovine serum, and 1% penicillin–streptomycin, as reported by Toda et al. (2021).

### 2.4 Macrophage modulation via siRNA targeting of S100A8/A9

RAW264.7 cells were seeded in six-well plates until they reached 60%–80% confluence. siRNA-S100A8 (50 nM) and siRNA-S100A9 (50 nM), purchased from Wuhan GeneCreate Biological Engineering Co., Ltd., were configured into the corresponding working solutions according to the instructions for RNAiMAX (13778150, Lipofectamine). The culture medium was replaced with complete medium following 6–8 h of treatment. When BMDM were induced to mature by M-CSF in a six-well plate, the targeted S100A8/A9 siRNA transfection, as mentioned above, was performed.

### 2.5 Overexpression plasmid synthesis and transfection

(i) Overexpression plasmid preparation: pCDH-S100A8-3×Flag-puroR and pCDH-S100A9-3×Flag-puroR plasmids were purchased from Wuhan Viraltherapy Technologies Co., Ltd. The Stbl3 *E. coli* containing the plasmids were amplified and then extracted for plasmids using an endotoxin-free plasmid extraction kit. (ii) Overexpression plasmid transfection: RAW264.7 cells were seeded in six-well plates and cultured until they reached 60%–80% confluence. The overexpression plasmids of S100A8 and S100A9 were transfected into RAW264.7 following the instructions for Lipo3000 (L3000015, Lipofectamine).

### 2.6 Lipopolysaccharide-induced macrophage polarization

When RAW264.7 and BMDM cells were grown to an appropriate state in six-well plates, they were treated with complete medium containing lipopolysaccharide (LPS, 100 ng/mL) for 12 h. The vehicle group was treated with equal phosphate-buffered saline (PBS) for 12 h. At the end of LPS treatment, the cells were used to perform relevant experiments, or the supernatant was collected after an additional 24 h.

### 2.7 Stimulation of recombinant protein S100A8/A9

TCMK-1 and mRTECs were passaged into six-well plates and grown to the appropriate confluence. The complete medium containing S100A8–S100A9 heterodimer recombinant protein (rS100A8/A9, HY-P71076, MedChemExpress) (1 μg/mL) was used to stimulate TCMK-1 cells for 24 h. Equal amounts of PBS were used for the vehicle group.

### 2.8 RNA extraction and quantitative reverse transcription-PCR

After treatment, all cells were washed twice with PBS. Cellular RNA was isolated using TRIzol reagent (15596018CN, Invitrogen) according to the manufacturer’s instructions. A total of 1 μg of RNA was reverse-transcribed into cDNA using the HiScript III RT SuperMix for qPCR (+ gDNA wiper) reagent (R323-01, Vazyme). Target gene mRNA expression levels were detected by quantitative real-time PCR using ChamQ Universal SYBR qPCR Master Mix (Q711-03, Vazyme) and a LightCycler® 480 real-time PCR system (Roche). *Actb* was used as the housekeeping gene for normalization. The ΔCt value was calculated by subtracting the Ct value of the housekeeping gene from the Ct value of the target gene. Specifically, the relative expression levels were calculated using the 2^−ΔΔCt^ method, where the ΔΔCt value was derived by comparing the ΔCt values of the experimental groups to those of the negative control group (NC) or the Vehicle control group (Vehicle). Primers were ordered from Genscript Biotech Corporation, and primer sequences are provided inSupplementary Table S1.

### 2.9 Western blot

The mouse kidneys were lysed into tissue homogenate using RIPA lysate (P0013B, Beyotime) and centrifuged at 12,000 rpm for 15 min at 4°C to obtain the protein supernatant. After measuring protein concentration by BCA protein quantification (23225, Thermo Scientific), the protein supernatant was mixed with the corresponding volume of SDS-PAGE sample-loading buffer (5×) (P0015L, Beyotime) and heated at 100°C for 10 min. The kidney proteins were electrophoresed using 15% SDS-PAGE gels (PG114, Epizyme) for 120 V for 60 min and transferred to a 0.22-μm PVDF membrane (ISEQ00010, Millipore) at 300 mA for 60 min. The membranes were blocked with 5% skimmed milk (configured using TBST buffer) for 2 h at room temperature, and then, the membranes were incubated with primary and secondary antibodies. The antibodies used included anti-β-actin (GB15001-100, Servicebio), anti-S100A8 (ab92331, Abcam), anti-S100A9 (ab105472, Abcam), Peroxidase AffiniPure™ goat anti-rabbit IgG (H + L) (AB_2307391, Jackson ImmunoResearch Laboratories, Inc.), and Peroxidase AffiniPure™ Goat anti-mouse IgG (H + L) (AB_10015289, Jackson ImmunoResearch Laboratories, Inc.). The chemiluminescence of protein bands was detected using the ECL Western Blotting Substrate (180-5001, Tanon). ACTB served as the internal control for normalization in the Western blot analysis. The intensity values of the target and internal control proteins were measured using ImageJ. The intensity values of the target protein were normalized to ACTB to represent its relative expression level.

### 2.10 Immunofluorescence

Mouse kidney tissues were paraffin embedded and cut into 5-μm sections. After dewaxing and hydration, antigens were retrieved by boiling the sections in sodium citrate buffer. The sections were permeabilized with 0.3% Triton-100X for 15 min at 4°C and blocked by 5% goat serum for 30 min at room temperature. The sections were then incubated with anti-S100A8, anti-S100A9, and anti-CD68 (ab955, Abcam) overnight at 4°C. The following day, the sections were incubated with the secondary antibodies, such as Goat Anti-Rabbit IgG H&L (Alexa Fluor 488) (ab150077, Abcam), Goat Anti-Mouse IgG H&L (Alexa Fluor 647) (ab150115, Abcam), and Rat Anti-Goat IgG H&L (Alexa Fluor 594) (ab150160, Abcam), for 1 h at room temperature away from light. DAPI (C1002, Beyotime) was used to incubate the sections for 15 min at room temperature away from light. Finally, the slices were sealed with a sealer. Images were taken with an Olympus FLUOVIEW FV3000.

### 2.11 Histological analysis and immunohistochemistry

Kidney tissues from mice (n = 3 per group) were fixed in 4% paraformaldehyde for 24–48 h, dehydrated, embedded in paraffin, and cut into 5-μm-thick sections. Sections were used for hematoxylin and eosin (H&E) stain and Masson stain to assess the extent of kidney injury in mice.

After deparaffinization and hydration, antigen retrieval was performed using sodium citrate. Paraffin sections were incubated using KIM-1 (30948-1-AP, Proteintech) and NGAL (30700-1-AP, Proteintech) as primary antibodies overnight at 4°C. Sections were incubated using horseradish peroxidase-labeled secondary antibodies for 2 h at room temperature and finally stained using diaminobenzidine (DAB). The dark brown marks observed under the microscope were positive signals.

### 2.12 Transcriptome sequencing

TCMK-1 cells were treated with culture supernatants from normal, S100A8/A9^hi^, and S100A8/A9^low^ M1-type RAW264.7 cells. RNA from TCMK-1 cells was extracted using TRIzol, and more than 1 μg of RNA was used for whole transcriptome RNA-sequencing analysis. P-value adjustment was applied using the Benjamini–Hochberg method (R package DESeq2). Bioinformatics analyses of the RNA-sequencing results were performed using “ggplot2” and “clusterprofiler” packages to visualize differentially expressed genes (DEGs) through volcano plots, Gene Ontology (GO) analysis, Kyoto Encyclopedia of Genes and Genomes (KEGG) analysis, and Gene Set Enrichment Analysis (GSEA) (Ito and Murphy, 2013; Wu T. et al., 2021).

### 2.13 Bioinformatics analysis

Six datasets related to AKI were retrieved from the NCBI Gene Expression Omnibus database (https://www.ncbi.nlm.nih.gov/geo/). The details of the six datasets are provided in Supplementary Table S2. GSE43974, GSE98622, and GSE226275 were transcriptome-sequencing databases, and gene annotations of these databases were performed in R software using “org.Mm.eg.db,” “org.Hs.eg.db,” and the GEO Platform. The “DESeq2” package was used for screening DEGs with P < 0.05 (Love et al., 2014). Venn diagrams were generated using the “VennDiagram” package. The intersection genes in these datasets were analyzed for KEGG enrichment (P < 0.05) via the database for annotation, visualization, and integrated discovery (DAVID). Heatmaps were plotted using an online platform for data analysis and visualization (https://www.bioinformatics.com.cn) (Tang et al., 2023a).

Analysis of GSE139506 was performed on the interactive website (https://research.cchmc.org/PotterLab/scIRI/). Expression levels of target genes in different cell clusters at different time points post-AKI were compared with those of the control group. “Seurat v5” was used to analyze GSE174219, GSE174220, and GSE199321 single-cell sequencing datasets. Dimension reduction operations [principal component analysis (PCA), t-distributed stochastic neighbor embedding (tSNE), uniform manifold approximation, and projection (UMAP)] were used to determine the location status of cells. RunHarmony function was used for de-batching of sequencing samples. “FindNeighbors” and “FindClusters” packages were used for cluster analysis. Identifications of each cell group were referred to the reported article. Marker genes of each cell cluster, which met the criterion of the expression exceeding 10% of cells per group and the average log-fold change >0.25, were selected using the Wilcoxon rank sum test within FindAllMarkers function. Expression levels of target genes in different cell clusters were visualized using the VlnPlot function.

### 2.14 Data analysis

Statistical analysis was performed using GraphPad Prism 8.3.0. Measurements that conformed to a normal distribution were expressed as mean ± SEM. Statistical comparisons between groups were conducted using t-test, one-way ANOVA, and other appropriate statistical methods. P < 0.05 was considered statistically significant.

## 3 Result

### 3.1 S100A8/A9 were significantly increased in the AKI mouse model

**FIGURE 1:**
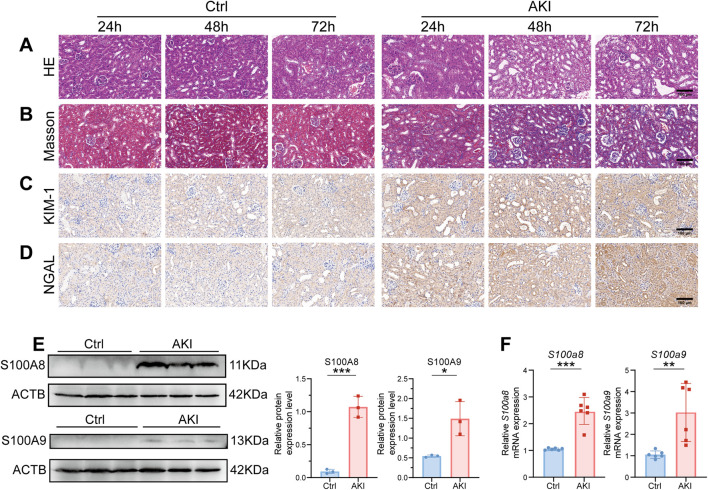
*S100a8/a9* were significantly increased in the AKI mouse model. **(A)** Representative H&E-stained images of mouse kidney tissues from the AKI group at 24, 48, and 72 h post-AKI, alongside the corresponding Ctrl group. **(B)** Representative Masson-stained images of mouse kidney tissues from the AKI group at 24, 48, and 72 h post-AKI, along with the corresponding Ctrl group. **(C)** Representative immunohistochemistry images of KIM-1 expression at 24, 48, and 72 h post-AKI, with the corresponding Ctrl group. **(D)** Representative immunohistochemistry images of NGAL expression at 24, 48, and 72 h post-AKI, with the corresponding Ctrl group. **(E)** Representative Western blot images and corresponding statistical analysis of S100A8/A9 expression in kidney tissues from the Ctrl and AKI groups. ACTB was used as the housekeeping gene for the normalization of protein expression levels across different genes. **(F)** mRNA expression levels of *S100a8/a9* in kidney tissues from the Ctrl and AKI groups. *Actb* was used as the housekeeping gene for the normalization of mRNA expression levels across different genes. AKI, acute kidney injury; Ctrl, control. Data are expressed as mean ± SEM. *P < 0.05, **P < 0.01, and ***P < 0.001.

In our previous study, plasma and urine levels of S100A8/A9 were elevated in ASA-AKI patients compared to those in non-ASA-AKI patients, as measured by ELISA (Wang et al., 2023). Moreover, S100A8/A9 plasma levels at 0 h post-surgery had a better predictive value than the urinary NGAL level and the Cleveland Clinic score (Wang et al., 2023). In this experiment, 24 h post-AKI onset, H&E-stained images of the AKI group revealed swollen renal tubular epithelial cells and exfoliated cells within the lumen, whereas in the Ctrl group, the cells appeared relatively intact (Figure 1A). Additionally, Masson-stained images revealed more severe fibrosis in the renal tissue of the AKI group than in the renal tissue of the Ctrl group (Figure 1B). The aforementioned changes progressively aggravated in the AKI group at 72 h after onset. Immunohistochemistry demonstrated that the expression levels of KIM-1 and NGAL were upregulated in the kidneys of all AKI groups (Figures 1C, D). These results indicated that the renal function of AKI mice was impaired. Western blot analysis revealed that the expression levels of S100A8/A9 were upregulated in the AKI group (24 h post-onset) compared to the Ctrl group (Figure 1E). The mRNA levels of *S100a8/a9* were markedly increased in the AKI group compared to the Ctrl group at 24 h after onset (Figure 1F). These results indicated a significant increase in S100A8/A9 expression in the AKI mouse model.

### 3.2 S100A8/A9 co-localized with macrophages in the AKI mouse model

**FIGURE 2:**
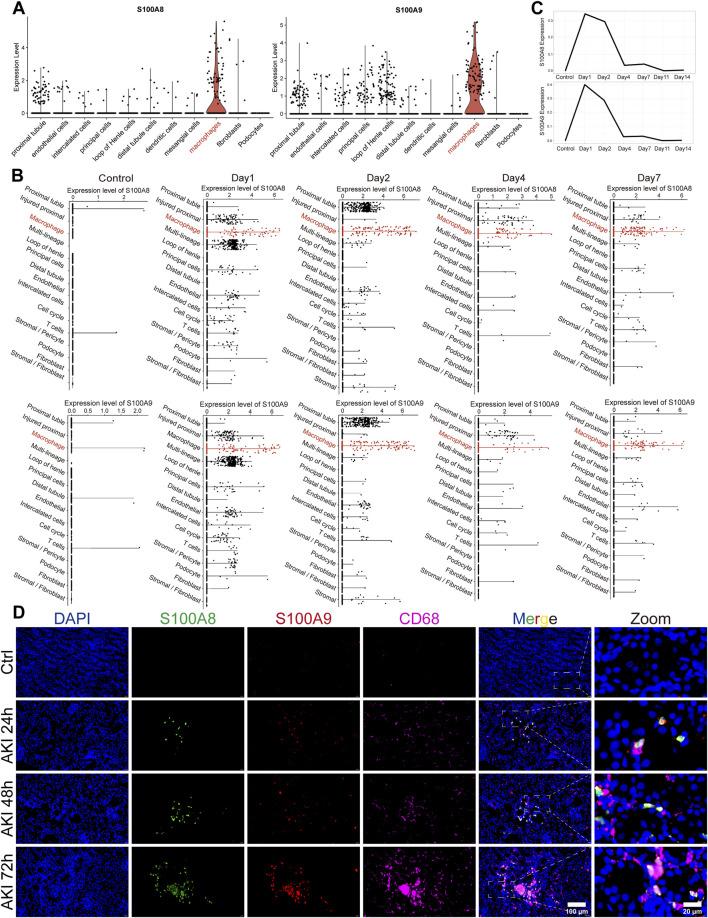
S100A8/A9^hi^ macrophages were increased during AKI. **(A)** Violin plots illustrate the expression levels of S100A8/A9 across different cell clusters in the sequencing data from AKI patients. **(B)** Scatterplots at different time points displayed expression levels of S100A8/A9 in various cell clusters from AKI mouse-sequencing data. **(C)** Line graphs showed S100A8/A9 expression changed at different time points in AKI mice. **(D)** Representative immunofluorescence images of renal sections from the Ctrl and AKI groups at 24, 48, and 72 h post-AKI. Sections were labeled with DAPI (blue), S100A8 (green), S100A9 (red), and CD68 (magenta) (n = 6). AKI, acute kidney injury; Ctrl, control.

To investigate the distribution levels of S100A8/A9 in different cell clusters, we selected several single-cell sequencing datasets for subsequent analysis. The GSE174219 and GSE174220 datasets consisted of kidney biopsies from healthy and AKI patients (Tang et al., 2023b). The GSE139506 dataset was an AKI murine model dataset (Rudman-Melnick et al., 2020). After performing clustering and dimensionality reduction analyses of GSE174219 and GSE174220 data, 11 cell clusters were identified using known marker genes. S100A8/A9 were highly expressed in the macrophage cluster of AKI patients (Figure 2A). The results at different time points in AKI mice were consistent with those observed in AKI patients (Figure 2B). The highest expression levels of S100A8/A9 in renal tissue of the AKI group were on the first day after onset, but they were barely expressed in the control group (Figure 2C). Immunofluorescence showed that S100A8/A9 proteins and CD68-labeled macrophages were scarcely detected in the Ctrl group, whereas S100A8 and S100A9 were notably increased and largely co-localized with macrophages at all time points in the AKI groups (Figure 2D).

**FIGURE 3:**
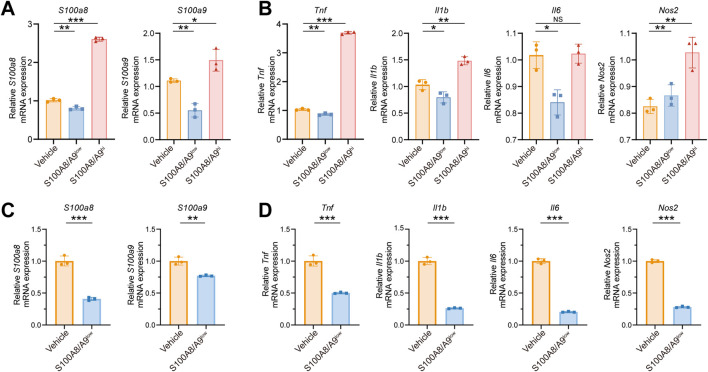
S100A8/A9 influence the M1 polarization of macrophages. **(A)** mRNA expression levels of *S100a8/a9* in the Vehicle, S100A8/A9^low^, and S100A8/A9^hi^ RAW264.7 groups. **(B)** mRNA expression levels of the classical inflammatory factors such as *Tnf, Il1b, Il6*, and *Nos2* in the Vehicle, S100A8/A9^low^, and S100A8/A9^hi^ RAW264.7 groups after LPS stimulation. **(C)** mRNA expression levels of *S100a8/a9* in the Vehicle group and S100A8/A9^low^ BMDM group. **(D)** relative mRNA expression levels of M1 polarization markers in the Vehicle group and S100A8/A9^low^ BMDM group after LPS stimulation. BMDM, mouse primary bone marrow-derived macrophage; S100A8/A9^hi^, high expression of S100A8/A9; S100A8/A9^low^, low expression of S100A8/A9. *Actb* was used as the housekeeping gene for the normalization of mRNA expression levels across different genes. Data are expressed as mean ± SEM. *P < 0.05, **P < 0.01, and ***P < 0.001.

We investigated the role of S100A8/A9 in macrophage polarization by dividing RAW264.7 cells into three groups before being treated with LPS: the Vehicle group (stimulated by PBS), the S100A8/A9 low-expression (S100A8/A9^low^) group (downregulated by siRNAs), and the S100A8/A9 high-expression (S100A8/A9^hi^) group (upregulated by overexpression plasmids). The expression levels of *S100a8/a9* were efficiently modulated by siRNAs and overexpression plasmids, respectively, compared to those of the Vehicle group (Figure 3A). Marker genes of M1-type polarization, such as *Tnf, Il6, Il1b*, and *Nos2*, exhibited upregulation of mRNA expression in the S100A8/A9^hi^ group compared to the Vehicle group, whereas the opposite trend was observed in the S100A8/A9^low^ group (Figure 3B). BMDM data further confirmed this. The mRNA levels of *S100a8/a9* were significantly reduced in BMDM by siRNAs targeting *S100a8/a9* compared to those of the Vehicle group (Figure 3C). The expression levels of M1 phenotypic markers were significantly reduced in the S100A8/A9^low^ group (Figure 3D). These results indicated that S100A8/A9 enhanced the M1-type polarization of macrophages and inflammatory response.

### 3.3 Transcriptome sequencing revealed that renal tubular epithelial cells were influenced by S100A8/A9^hi^ and S100A8/A9^low^ macrophages

**FIGURE 4:**
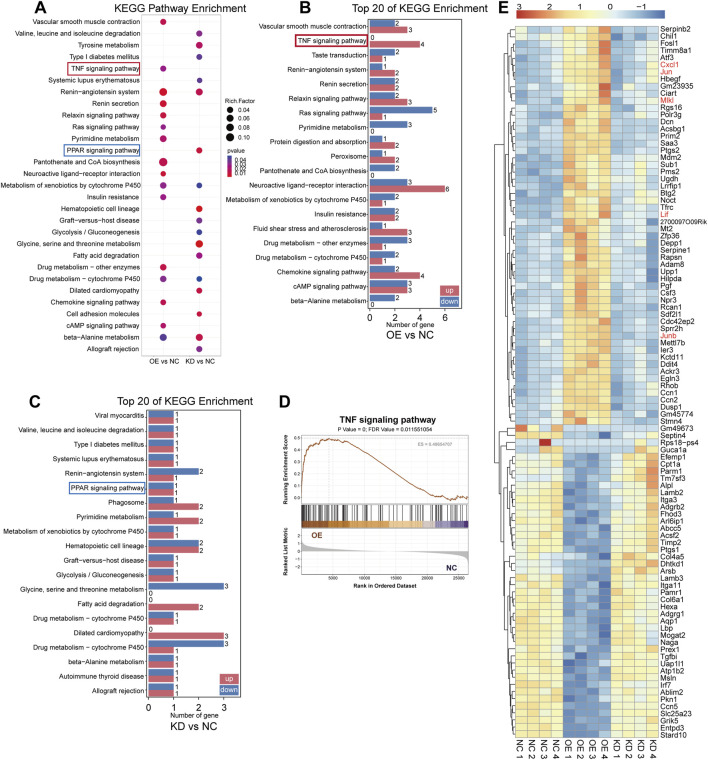
Transcriptome sequencing revealed that renal tubular epithelial cells were influenced by S100A8/A9^hi^ and S100A8/A9^low^ macrophages **(A)** The bubble plots showed the significantly enriched KEGG pathways (P < 0.05) from the OE group versus the NC group and the KD group versus the NC groups, with an absence of bubbles indicating P ≥ 0.05. **(B)** Among the top 100 DEGs in the OE group compared to the NC group, the bar graph illustrates the number of upregulated and downregulated genes in the relevant signaling pathways. **(C)** Among the top 100 DEGs in the KD group compared to the NC group, the bar graph shows the number of upregulated and downregulated genes in the relevant signaling pathways. **(D)** The GSEA plot shows the enrichment of TNF signaling pathways in the OE group. **(E)** The heatmap displays the expression levels of the top 100 DEGs in the OE group versus the NC group and the KD group versus the NC group. Genes associated with the TNF pathway are marked in orange font. DEGs, differentially expressed genes; KEGG, Kyoto Encyclopedia of Genes and Genomes; KD, knockdown; NC, negative control; OE, overexpression.

To explore the underlying mechanisms of ASA-AKI, we simulated the pathological changes by inducing damage to renal tubular epithelial cells using macrophages. Specifically, after LPS treatment, supernatants from RAW264.7 of the Vehicle, S100A8/A9^low^, and S100A8/A9^hi^ groups were collected after an additional 24 h. These supernatants were then used to treat TCMK-1 cells in the negative control (NC), knockdown (KD), and overexpression (OE) groups, respectively. Subsequently, transcriptome sequencing was performed on the TCMK-1 cells from three groups. Compared to the NC group, DEGs in the OE group showed that 162 genes were upregulated and 150 genes were downregulated, with *Saa3, Dcn, Sprr2h*, and *Cxcl1* being the most significantly upregulated (Supplementary Figure S1A). GO enrichment analysis revealed significant enrichment of renal water transport and nitric oxide transport functions in the OE group (Supplementary Figure S1C). KEGG analysis further showed the enrichment of TNF, RAS, chemokine, and cAMP signaling pathways in the OE group, as opposed to the NC group (Figure 4A). The bar graph shows the number of upregulated and downregulated genes in the top 20 KEGG pathways significantly enriched in the OE group, with the number of upregulated genes in the TNF pathway being significantly higher than the number of downregulated genes (Figure 4B). The GSEA plot shows the enrichment of TNF signaling pathways in the OE group, with a threshold of P < 0.05 and FDR <0.25 (Figure 4D; Supplementary Figure S1D). These findings suggest that the physiological functions of TCMK-1 cells were altered and that the activation of inflammatory signaling pathways in TCMK-1 cells was influenced by secretion from S100A8/A9^hi^ RAW264.7.

Additionally, compared to the NC group, DEGs in the KD group included 103 upregulated genes and 95 downregulated genes in TCMK-1 (Supplementary Figure S1B). GO enrichment analysis showed that TCMK-1 cells in the KD group exhibited minimal impact on renal water transport and nitric oxide transport functions (Supplementary Figure S1C). Meanwhile, KEGG analysis revealed that the KD group was primarily enriched in the PPAR pathway compared to the NC group (Figure 4A). The bar graph shows the number of upregulated and downregulated genes in the top 20 KEGG pathways significantly enriched in the KD group, with the number of upregulated and downregulated genes in the PPAR pathway being equal (Figure 4C). The heatmap showed the top 100 genes with the most significant fold changes in expression levels from the three subgroups. The TNF pathway-related genes, such as *Jun, Junb, Cxcl1*, and *Lif*, were upregulated in the OE group on the heatmap, whereas no significant changes were observed in the NC and KD groups (Figure 4E). According to the literature, activation of the TNF pathway induced the release of inflammatory mediators, recruited inflammatory cells, and regulated necrosis and apoptosis (Kalliolias and Ivashkiv, 2016). In contrast, the PPAR pathway has an inhibitory effect on the inflammatory response (Liu et al., 2020; Ishtiaq et al., 2022; Li et al., 2023). Therefore, S100A8/A9 may contribute to ASA-AKI by activating the TNF pathway-mediated inflammatory response, which injures renal tubular epithelial cells.

### 3.4 Multiple transcriptome analysis revealed that the TNF pathway was activated in AKI patients and a murine model

**FIGURE 5:**
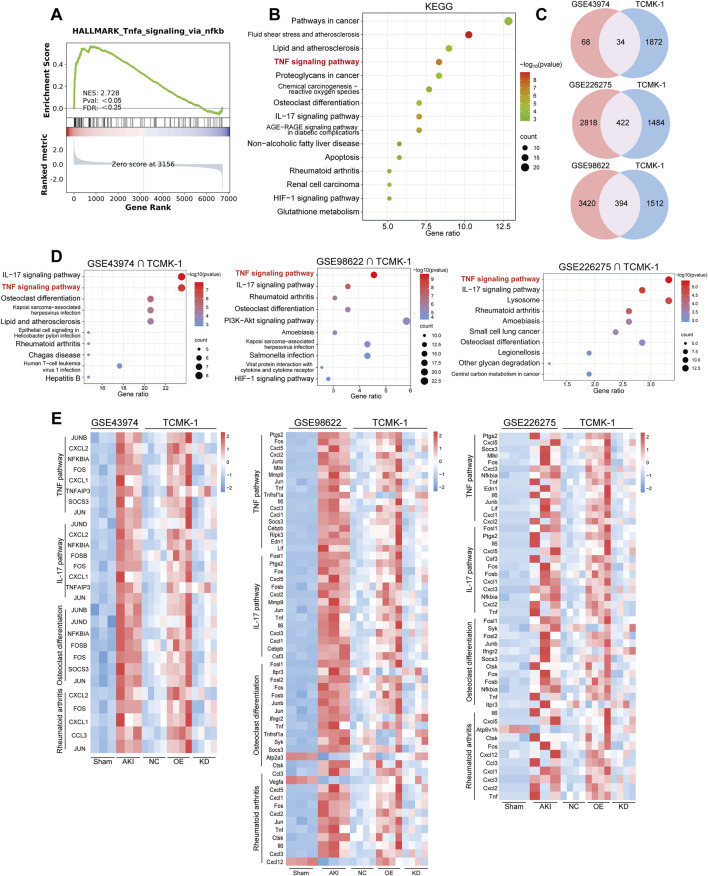
The TNF pathway was activated in multiple transcriptomes of AKI patients and the murine model. **(A)** The GSEA plot shows the enrichment of the TNF pathways in damaged renal tubular epithelial cells from the urine of ASA-AKI patients compared to normal renal tubular epithelial cells. **(B)** The bubble plot displays the KEGG enrichment analysis of DEGs related to inflammation in damaged renal tubular epithelial cells compared to normal renal tubular epithelial cells, both derived from the urine of ASA-AKI patients. **(C)** The Venn diagram shows the intersection of DEGs in TCMK-1 (P < 0.05, q value <0.35) and other datasets (P < 0.05) separately. **(D)** The bubble plot displays the top 10 KEGG pathways enriched by shared DEGs from those intersection clusters. **(E)** The heatmap exhibits the expression level of genes in representative KEGG pathways in those intersection clusters. AKI, acute kidney injury; ASA-AKI, acute type A aortic dissection-associated acute kidney injury; DEGs, differentially expressed genes; GSEA, Gene Set Enrichment Analysis; NC, negative control; OE, overexpression; KD, knockdown.

To further investigate renal tubular epithelial cell injury in ASA-AKI patients, we analyzed a human urine single-cell sequencing dataset (GSE199321) to assess cellular changes in these patients (Klocke et al., 2022). GSEA revealed the upregulation of TNF signaling pathways in renal tubular epithelial cells with a high expression of injury markers (case group) compared to those with a low expression (control group), with a threshold of P < 0.05 and FDR <0.25. (Figure 5A). KEGG analysis further revealed that inflammation-associated DEGs were predominantly enriched in the TNF signaling pathway in the case group compared to the control group (Figure 5B). These findings further highlighted the important role of the TNF signaling pathway in the injury process of renal tubular epithelial cells in ASA-AKI.

Additionally, we cross-analyzed the TCMK-1 sequencing results with a dataset from AKI patients (GSE43974) and two datasets from AKI mice (GSE98622 and GSE226275) to investigate the correlation between *in vitro* and *in vivo* lesions (Damman et al., 2015; Liu et al., 2017; Jia et al., 2023). A total of 1905 DEGs were screened from the NC, OE, and KD groups in TCMK-1 sequencing based on P < 0.05 and q-value (same as correction for P-value) < 0.35. DEGs from the GSE43974 dataset (P < 0.05) were intersected with the DEGs from the TCMK-1 sequencing result to obtain 34 common DEGs (Figure 5C). DEGs from GSE98622 and GSE226275 (P < 0.05) were intersected with the DEGs from TCMK-1 sequencing results to select 394 and 422 shared DEGs, respectively (Figure 5C). These shared DEGs from three intersection clusters were all enriched in the TNF pathway by KEGG pathway enrichment (Figure 5D). Additionally, the IL-17 signaling pathway, osteoblast differentiation, and rheumatoid arthritis were common enrichment pathways in three intersection clusters (Figure 5D). The genes from the TNF pathway and other common enrichment pathways, visualized by heatmap, were predominantly upregulated in the AKI group across three datasets and in the OE group from the TCMK-1 dataset (Figure 5E). These findings suggested that the activation of the TNF pathway and other inflammation-related pathways in renal tubular epithelial cells, triggered by S100A8/A9^hi^ macrophages, may play a crucial role in the development of ASA-AKI.

### 3.5 Recombinant protein S100A8/A9 activated the TNF signaling pathway in renal tubular epithelial cells

**FIGURE 6:**
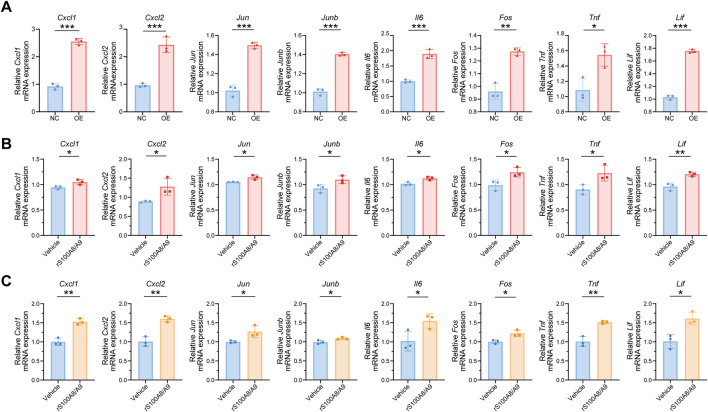
S100A8/A9 can activate the TNF signaling pathway in impaired renal tubular epithelial cells as secretory proteins **(A)** mRNA expression levels of *Cxcl1, Cxcl2, Jun, Junb, Il6, Fos, Tnf*, and *Lif* in TCMK-1 cells from the NC and OE groups. **(B)** mRNA expression levels of *Cxcl1, Cxcl2, Jun, Junb, Il6, Fos, Tnf*, and *Lif* in TCMK-1 cells after being treated with Vehicle or rS100A8/A9 (1 μg/mL) for 48 h. **(C)** mRNA expression levels of *Cxcl1, Cxcl2, Jun, Junb, Il6, Fos, Tnf*, and *Lif* in mRTECs after stimulation with Vehicle or rS100A8/A9 for 48 h. NC (negative control) represents TCMK-1 cells stimulated with supernatants from normal M1-type RAW264.7. OE (overexpression) represents TCMK-1 cells stimulated with supernatants from S100A8/A9^hi^ M1-type RAW264.7. NS, no significance; rS100A8/A9, recombinant S100A8/A9; mRTECs, primary mouse renal tubular epithelial cells. *Actb* was used as the housekeeping gene for the normalization of mRNA expression levels across different genes. Data are expressed as mean ± SEM. *P < 0.05, **P < 0.01, and ***P < 0.001.

To validate the sequencing results, 28 genes from the top 100 DEGs were selected. These genes were mainly enriched in key inflammation-related signaling pathways, such as the TNF signaling pathway, IL-17 signaling pathway, and osteoblast differentiation. The expression levels of these genes were assessed in TCMK-1 cells from both the NC and OE groups, which were stimulated with supernatants from M1-type RAW264.7 cells and S100A8/A9^hi^ M1-type RAW264.7 cells, respectively (Figure 6A; Supplementary Figure S2). The expression levels of TNF pathway-related genes, including *Cxcl1, Cxcl2, Jun, Junb, Il6, Fos, Tnf*, and *Lif*, were increased in the OE group compared to those in the NC group, which were consistent with the analysis results of multiple transcriptomic datasets (Figure 6A). These results suggested that S100A8/A9^hi^ RAW264.7 cells primarily injured TCMK-1 by activating the TNF pathway.

S100A8/A9 can influence both synthetic cells and surrounding cells through autocrine and paracrine mechanisms as a secreted protein (Song and Struhl, 2021). Therefore, we inferred that S100A8/A9 had a direct effect on renal tubular epithelial cells as secretory proteins. We next stimulated TCMK-1 with S100A8-S100A9 heterodimer recombinant protein (rS100A8/A9, 1 μg/mL) for 48 h, and the Vehicle group was treated with an equal volume of PBS under the same conditions. The expression levels of *Cxcl1, Cxcl2, Jun, Junb, Il6, Fos, Tnf*, and *Lif* were increased in the rS100A8/A9 group compared to the Vehicle group (Figure 6B). The gene expression involved in the TNF signaling pathway was also upregulated in mRTECs after rS100A8/A9 stimulation (Figure 6C). This result is consistent with the findings from the TCMK-1 cell experiments and further confirms the role of S100A8/A9 in activating the TNF pathway. Thus, S100A8/A9, as secreted proteins, contribute to the injury of renal tubular epithelial cells in ASA-AKI by activating the TNF pathway.

## 4 Discussion

In this study, we observed that the expression levels of S100A8/A9 were increased in the kidneys of AKI mice, which were consistent with our previous findings that S100A8/A9 increased in both the plasma and urine of ASA-AKI patients at 0 h after ATAAD surgery (Wang et al., 2023). S100A8/A9 were highly expressed in macrophage clusters in both AKI patients and murine models. Furthermore, we found that S100A8/A9^hi^ RAW264.7 cells promoted polarization toward the M1 phenotype, whereas downregulation of S100A8/A9 had the opposite effect. The analysis of transcriptomic data from TCMK-1 cells in this study, along with sequencing data from public datasets of ASA-AKI patients, AKI patients, and murine models, revealed that the TNF signaling pathway is activated in damaged renal tubular epithelial cells during AKI. Moreover, rS100A8/A9 upregulated the TNF pathway in TCMK-1 cells. Therefore, in ASA-AKI patients, S100A8/A9 may activate the TNF signaling pathway in renal tubular epithelial cells by modulating macrophage polarization and acting as secretory proteins.

According to previous studies from our center and the results of this study, S100A8/A9 were found to be increased in both ASA-AKI patients and AKI mouse models (Wang et al., 2023). Yao et al. found a significant correlation between the expression levels of S100A8 and S100A9 in kidneys and the severity of AKI in patients by renal biopsy (Yao et al., 2022). Furthermore, Pruenster et al. (2016) previously summarized that S100A8/A9 can trigger pro-inflammatory effects by binding to glycosaminoglycans (GAGs), the receptor for advanced glycosylation end-products (RAGE), and Toll-like receptor 4 (TLR4) on the cytomembrane. *Jun* and *Junb*, members of the activator protein 1 (AP-1) transcription factor family, were upregulated in the sequencing data of TCMK-1 cells treated with S100A8/A9^hi^ M1-type RAW264.7 culture supernatants, as well as in AKI mouse models and patients, consistent with previous reports (Liu et al., 2017; Gerhardt et al., 2021). The AP-1 transcription factor family, which consists of Jun, Fos, ATF, Maf, and their subtypes, is involved in TNF, RAGE, and TLR4 signaling pathways (Liu et al., 2017; Bejjani et al., 2019; Wu Z. et al., 2021; Ji et al., 2019; Inciarte-Mundo et al., 2022). As a ligand for RAGE and TLR4 receptors, S100A8/A9 activate transcription factors such as AP-1, NF-κB, and CREB1 via the MyD88 pathway, initiating downstream inflammatory pathways that intertwined with the inflammatory response triggered by the TNF pathway, thereby forming a complex network (Inciarte-Mundo et al., 2022; Aggarwal, 2003). Moreover, AP-1 transcription factors have been reported to play a role in acute kidney injury, with single-cell sequencing indicating their involvement in the injury response of proximal tubular cells (Yu et al., 2023; Shen et al., 2016; Gerhardt et al., 2021). Future experiments will explore whether AP-1 acts as a mediator linking S100A8/A9 to the TNF signaling pathway in damaged renal tubular epithelial cells, with a focus on understanding the molecular mechanisms involved. Additionally, this study found that rS100A8/A9 increases the expression of TNF pathway-related genes in both TCMK-1 cells and mRTECs, consistent with previous reports showing that recombinant S100A8/A9 promote the expression of TNF pathway-related genes in renal mesangial and THP-1 cells (Pepper et al., 2015; Wang et al., 2015; Simard et al., 2013). Therefore, the TNF signaling pathway was a major inflammatory injury pathway in the interaction of macrophages with renal tubular epithelial cells.

There were some limitations in this article. First, the invasiveness of renal puncture biopsy and the critical condition of patients with ATAAD limit renal biopsy as a routine test in the clinic (Klocke et al., 2022). Moreover, the AKI murine model of unilateral kidney ischemia-reperfusion cannot adequately resemble the pathological process of ASA-AKI in humans, such as ATAAD formation, administration of contrast agents, using nephrotoxic drugs, and surgery under cardiopulmonary bypass, among others (Mehran et al., 2019; Zhang et al., 2020; Helgason et al., 2021; Nielsen et al., 2011). Therefore, it is necessary to explore animal models that are more suitable for the pathogenesis of ASA-AKI in the future. In addition, BMDM have the advantage of being more stable in gene expression and more accurately reflecting cellular changes during the disease process (Herb et al., 2024). However, their application is limited by the difficulty of transient genetic modification. In contrast, RAW264.7 cells, with their strong proliferative capacity and ease of transient genetic modification, are more widely used in experiments (Herb et al., 2024). Furthermore, the analysis of S100A8/A9 expression in neutrophils in AKI tissue was lacking here because the original literature cited for single-cell sequencing did not include a neutrophil cluster. This limitation may be related to the fact that neutrophil transcript levels were low compared to those in other cells and degraded rapidly in isolated tissues, making them susceptible to filtering during the processing of the sequencing data (Hay et al., 2018). Finally, previous studies have demonstrated that not only S100A8/A9 promotes the synthesis of TNF-α but TNF-α, in turn, can also enhance the production of S100A8/A9 in certain diseases (Chen et al., 2023; Jiang et al., 2024; Nicaise et al., 2017). Therefore, the underlying mechanism by which S100A8/A9 from macrophages affect the TNF signaling pathway in renal tubular epithelial cells in ASA-AKI patients needs to be further confirmed in future studies using relevant knockout mouse models.

In addition to the well-known role of S100A8/A9 as heterodimers in pro-inflammatory responses, there is growing attention on its ability to exert anti-inflammatory effects as tetramers in inflammatory processes (Xu et al., 2024). The tetrameric form of S100A8/A9 transforms from the dimeric form, influenced by factors such as extracellular metal ion concentration, reactive oxygen species (ROS) levels, and pH, which conceals the TLR4 binding site (Russo et al., 2022; Vogl et al., 2018). Studies have shown that the regulatory effect of S100A8/A9 on the activity of tumor cells depends on their concentration: at high concentrations, S100A8/A9 inhibit tumor cell growth and promote their apoptosis, whereas at low concentrations, they exhibit the opposite effect (Kwon et al., 2013; Ghavami et al., 2008). The S100A8/S100A9 tetramer modulates the immune response in skin granuloma and irritant contact dermatitis models by regulating the basal migration of monocytes through specific interactions with CD69 (Russo et al., 2022). Furthermore, a high-affinity peptide binder for S100A8/A9 has been developed, which can specifically bind to the tetrameric form of S100A8/A9, offering a more convenient, stable, and sensitive detection method (Díaz-Perlas et al., 2023). In future studies, we aim to explore the mechanism of S100A8/A9 through a more comprehensive perspective.

Given the promising potential of S100A8/A9-targeted therapies, further strategies have been explored to enhance their therapeutic effects. Small-molecule inhibitors of S100A8/A9, such as paquinimod and ABR-238901, can disrupt the interaction between S100A8/A9 and RAGE or TLR4 (Pruenster et al., 2016). Nanoparticle delivery systems using nanoparticles or liposomes can effectively deliver targeted S100A8/A9 antibodies or inhibitors, whereas gene-silencing techniques like siRNA can reduce S100A8 and S100A9 expression at both the mRNA and protein levels (Shang et al., 2024; Lu et al., 2024a). Moreover, existing research has demonstrated that S100A8/A9-neutralizing antibodies exhibit promising therapeutic effects in animal models of myocardial infarction, idiopathic pulmonary fibrosis, ischemia-reperfusion-induced lung injury, melanoma, and atopic dermatitis (Li et al., 2019; Araki et al., 2021; Nakata et al., 2022; Kinoshita et al., 2019; Gohara et al., 2025). Renal tubular epithelial cells are the primary target of damage during AKI, and targeted drugs face limitations in size and molecular weight due to the glomerular filtration barrier, which can affect drug delivery efficiency (Li et al., 2024; Shang et al., 2024). However, engineered targeted drug delivery systems, such as nanoparticles, peptides, and antibodies, could effectively overcome these barriers to target the kidneys (Shang et al., 2024; Lu et al., 2024b). These strategies highlight the promising potential of S100A8/A9-targeted therapy in treating AKI; however, further research is needed to validate their safety, efficacy, and stability.

## 5 Conclusion

In the AKI murine model, the expression levels of S100A8/A9 and their co-localization with macrophages were increased. The overexpression of S100A8/A9 in M1-type macrophages strongly enhanced polarization toward the M1 phenotype. S100A8/A9 were found to activate the TNF pathway, leading to impairment of renal tubular epithelial cells through the modulation of macrophage polarization and as secretory proteins. These findings suggest that S100A8/A9 may play a significant role in the pathogenesis of ASA-AKI.

## Data Availability

The original contributions presented in this study are publicly available. The transcriptome sequencing data of TCMK-1 has been provided as a supplementary list in the Supplementary Material. Additionally, the following sequencing datasets are available for download from GEO Datasets: GSE43974, GSE98622, GSE226275, GSE174219, GSE174220, GSE139506, and GSE199321.
