# Supplementary material for: S100A8/A9 high-expression macrophages mediate renal tubular epithelial cell damage in acute kidney injury following acute type A aortic dissection surgery
Source: Front Mol Biosci. 2025 Apr 9;12:1530741. doi: 10.3389/fmolb.2025.1530741 (PMC12015165; doi:10.3389/fmolb.2025.1530741)
Supplement: Supplementary file 1 [file Presentation1.pdf]

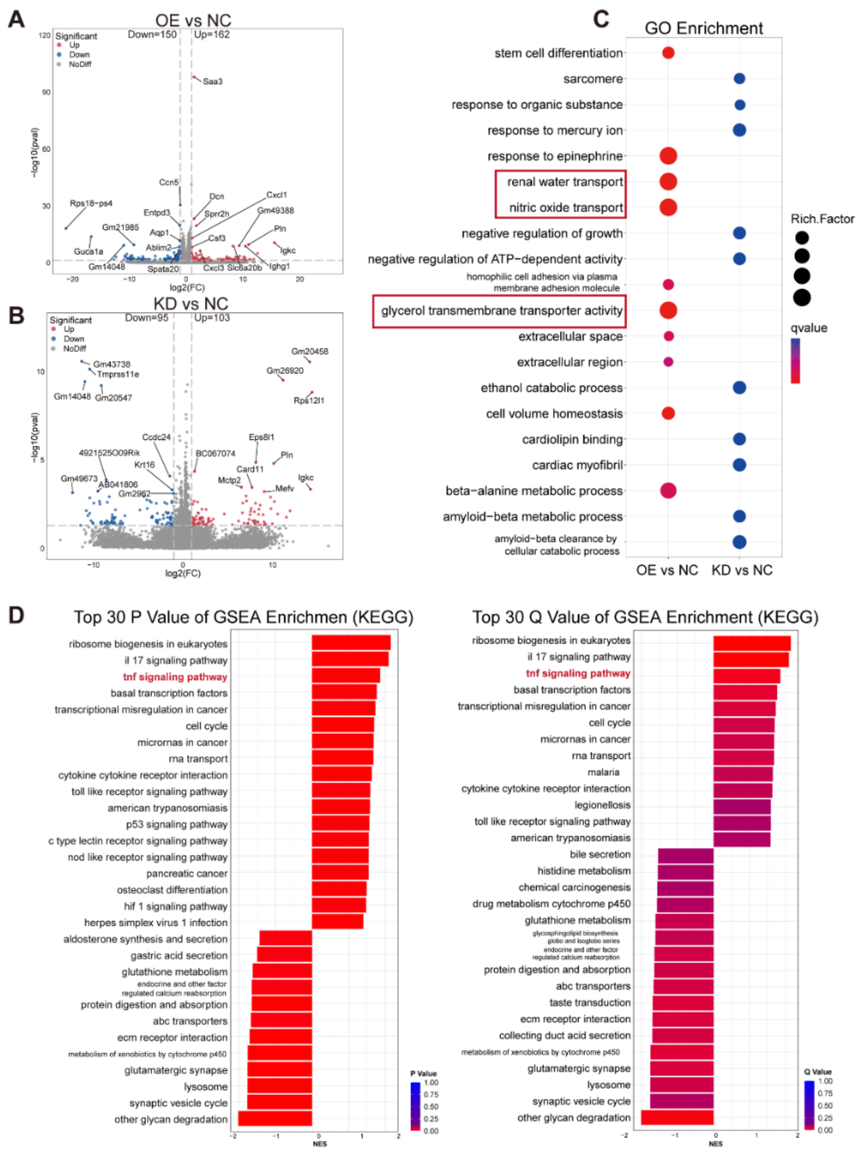

Figure S1: Partial results of transcriptional sequencing data from the TCMK-1 cells

(A) The volcano plot illustrated the DEGs in the OE group compared to the NC group. (B) The volcano plot showed the DEGs in the KD group versus the NC group. DEGs satisfied with |log2(fc)| > = 1 and P < 0.05 in (A-B). (C) The bubble plots represented the top 10 significantly enriched GO terms (P < 0.05) from OE versus NC and KD versus NC, with absence of bubbles indicating P ≥ 0.05. (D) The bar graph presented the enrichment of the top 30 gene sets ranked by p-values and q-values in the KEGG classification. DEGs, differentially expressed genes; KEGG, Kyoto Encyclopedia of Genes and Genomes; KD, knockdown; NC, negative control; OE, overexpression.

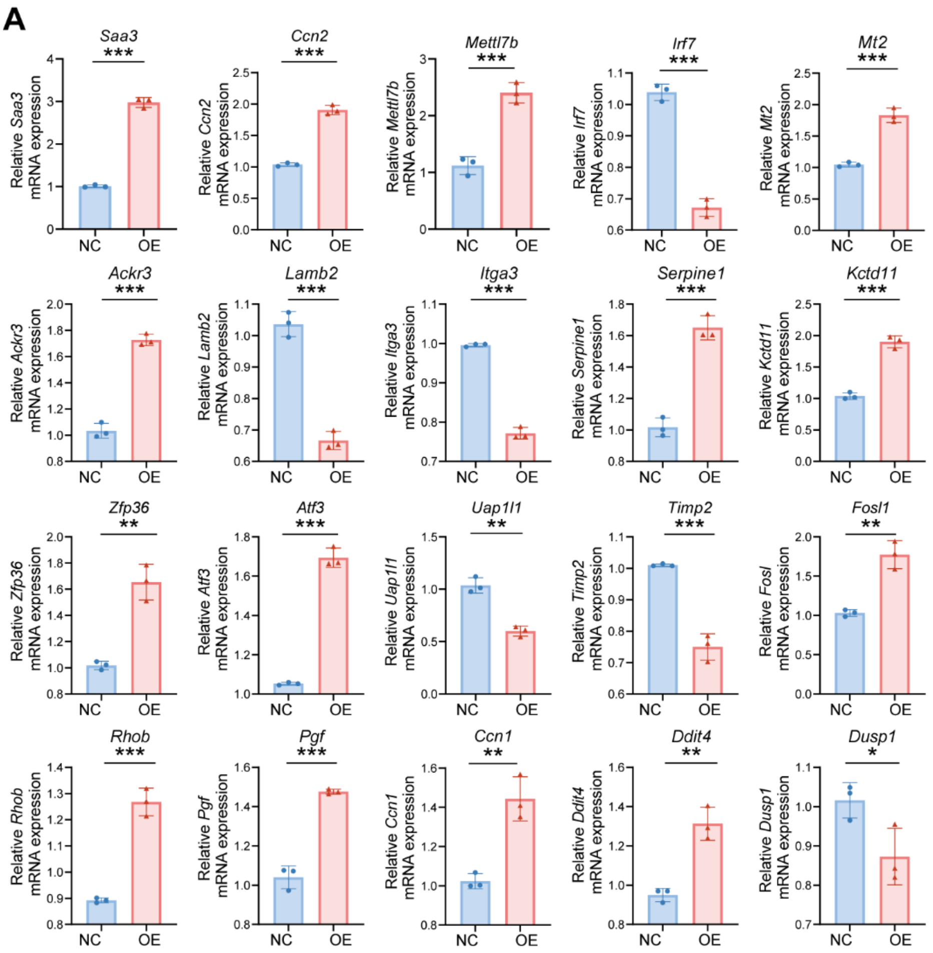

Figure S2: Validation of sequencing results in renal tubular epithelial cells

(A) The mRNA expression levels of 20 of the top 100 most significantly differentially expressed genes were measured in TCMK-1 cells stimulated by supernatants from M1-type RAW264.7 cells and S100A8/A9^{hi} M1-type RAW264.7 cells (n = 3). Data are expressed as mean ± SEM. *P < 0.05, **P < 0.01, ***P < 0.001.

Supplementary Table S1: The primers used in quantitative PCR

Gene Name (Mouse)	Sequence
S100a8	F: AAATCACCATGCCCTCTACAAG
	R: CCCACTTTTATCACCATCGCAA
S100a9	F: CCAACAAAGCACCTTCTCAG
	R: TTGCCATCAGCATCATACAC
Nos2	F: GGCTGGGTTTAGGGCTGTG
	R: CTGAGGGTGTCGTAGGTGATG
Il-6	F: GTTGCCTTCTTGGGACTGATGTTG
	R: CTGGTCTGTTGTGGGTGGTATCC
Tnf	F: CCCTCACACTCAGATCATCTTCT
	R: GCTACGACGTGGGCTACAG
Il1b	F: GCAACTGTTCCTGAACTCAACT
	R: ATCTTTTGGGGTCCGTCAACT
Jun	F: CCTTCTACGACGATGCCCTC
	R: GGTTCAAGGTCATGCTCTGTTT
Cxcl1	F: CTGGGATTCACCTCAAGAACATC
	R: CAGGGTCAAGGCAAGCCTC
Fosl1	F: ATGTACCGAGACTACGGGGAA
	R: CTGCTGCTGTCGATGCTTG
Junb	F: TCACGACGACTCTTACGCAG
	R: CCTTGAGACCCCGATAGGGA
Lif	F: ATTGTGCCCTTACTGCTGCTG
	R: GCCAGTTGATTCTTGATCTGGT
Cxcl2	F: CCAACCACCAGGCTACAGG
	R: GCGTCACACTCAAGCTCTG
Saa3	F: TGCCATCATTCTTTGCATCTTGA
	R: CCGTGAACTTCTGAACAGCCT
Cxcl2	F: CCAACCACCAGGCTACAGG
	R: GCGTCACACTCAAGCTCTG
Mettl7b	F: GATGCCCTGGTTCTATTTCTGC
	R: CATCTTTTTGTAGGACCTGGCT
Irf7	F: GAGACTGGCTATTGGGGGAG
	R: GACCGAAATGCTTCCAGGG
Mt2	F: GCCTGCAAATGCAAACAATGC
	R: AGCTGCACTTGTCGGAAGC
Ackr3	F: AGCCTGGCAACTACTCTGACA
	R: GAAGCACGTTCTTGTTAGGCA
Lamb2	F: GAACTTCGCTTGGGCCTACTT
	R: GGTGGCTGGATAGCAGCTT

Itga3	F: CCTCTTCGGCTACTCGGTC<br>R: CCGGTTGGTATAGTCATCACCC
Serpine1	F: TTCAGCCCTTGCTTGCCTC<br>R: ACACTTTTACTCCGAAGTCGGT
Kctd11	F: CTGGGGGCCATGTTTAGGG<br>R: AATTGAGGATGTGCCGGAAAG
Zfp36	F: CCACCTCCTCTCGATACAAGA<br>R: GCTTGGCGAAGTTCACCCA
Atf3	F: GAGGATTTTGCTAACCTGACACC<br>R: TTGACGGTAACTGACTCCAGC
Uap1l1	F: CATCTCCTGCGCTTCTATGCC<br>R: CGTGTCTCTTGGTCACAGC
Timp2	F: TCAGAGCCAAAGCAGTGAGC<br>R: GCCGTGTAGATAAACTCGATGTC
Fosl1	F: ATGTACCGAGACTACGGGGAA<br>R: CTGCTGCTGTCGATGCTTG
Rhob	F: GTGCCTGCTGATCGTGTTCA<br>R: CCGAGAAGCACATAAGGATGAC
Pgf	F: TCTGCTGGGAACAACTCAACA<br>R: GTGAGACACCTCATCAGGGTAT
Ccn1	F: CTGCGCTAAACAACTCAACGA<br>R: GCAGATCCCTTTCAGAGCGG
Ddit4	F: CAAGGCAAGAGCTGCCATAG<br>R: CCGGTACTTAGCGTCAGGG
Dusp1	F: GTTGTTGGATTGTCGCTCCTT<br>R: TTGGGCACGATATGCTCCAG

Supplementary Table S2: Characteristics of datasets.

GEO accession	Platform	Species	Contains	Country	Year	Contributor
GSE43974	GPL10558	Homo	Three kidney tissue biopsies from the T1 time point were randomly selected as the NC group and three kidney tissue biopsies from the T3 time point were randomly selected as the AKI group, and the sequencing results were used for this bioinformatic analysis.	Netherlands	2013	Jeffrey Damman
GSE98622	GPL13112	Mus	Samples from 1 day after AKI and the corresponding sham-operated group samples were selected for bioinformatics analysis.	American	2017	Jing Liu
GSE226275	GPL24247	Mus	3 AKI samples and 3 sham samples were used for bioinformatics analysis.	China	2023	Meng Jia
GSE174219	GPL20795	Homo	A sample from kidney biopsies of a healthy patient was used for analysis.	China	2022	YONG ZHONG
GSE174220	GPL20795	Homo	A sample from kidney biopsies of an AKI patient was used for analysis.	China	2022	YONG ZHONG
GSE139506	GPL17021	Mus	A Control sample and several AKI samples at different time points were selected for analysis.	American	2020	Steve Potter
GSE199321	GPL20301	Homo	2 patients of acute type A aortic dissection associated acute kidney injury were selected for analysis.	Germany	2022	Kim S, Klocke J, Boltengagen A, Skopnik C, Kocks C, Eckardt K, Rajewsky N, Enghard P
